# BRICS na gestão da pandemia de COVID-19: um estudo comparativo com foco nas ações de distanciamento social e vacinação entre as nações do bloco

**DOI:** 10.1590/0102-311XPT069024

**Published:** 2025-06-09

**Authors:** Thalyta Cássia de Freitas Martins, Raphael Mendonça Guimarães, Adelyne Maria Mendes Pereira

**Affiliations:** 1 Fundação Oswaldo Cruz, Rio de Janeiro, Brasil.; 2 Universitat de les Illes Balears, Illes Balears, España.

**Keywords:** Sistemas de Saúde, Política de Saúde, COVID-19, Vacinação, Health Systems, Health Policy, COVID-19, Vaccination, Sistemas de Salud, Política de Salud, COVID-19, Vacunación

## Abstract

O objetivo foi analisar, de forma comparada, as medidas de distanciamento social e vacinação implementadas pelos países do Brics em resposta à pandemia da COVID-19. Realizou-se estudo exploratório ancorado nas contribuições do institucionalismo histórico e do método histórico-comparado em ciências sociais. Dois eixos foram priorizados: as medidas de distanciamento social e de vacinação. Parte dos países do BRICS apresentaram características semelhantes, como a adoção de medidas de distanciamento social pouco extensas, flexíveis e sob coordenação descentralizada (com exceção da China); fragilidade das medidas de auxílio social (Brasil, Índia e África do Sul); e o uso de recursos biotecnológicos (presentes na China e na Rússia). A respeito da vacinação, três aspectos se assemelham: início tardio (exceto na China e na Rússia); cobertura vacinal (esquema completo) inferior a 70% da população em dezembro de 2022 (exceção para China e Brasil); e hesitação vacinal. As respostas adotadas pelos países do BRICS em termos de distanciamento social e vacinação contra COVID-19 foram diversas e relacionadas a fatores políticos, sociais e econômicos, que condicionaram a implementação de tais medidas.

## Abstract (en)

This study aimed to comparatively analyze the social distancing and vaccination measures BRICS countries implemented in response to the COVID-19 pandemic. This exploratory study is anchored in the contributions of historical institutionalism and the historical-comparative method in Social Sciences. This research prioritized two axes: social distancing and vaccination measures. Some BRICS countries showed similar characteristics, such as loose, flexible, social distancing measures under decentralized coordination (except for China); frail social assistance measures (Brazil, India, and South Africa); and biotechnological resources (in China and Russia). Regarding vaccination, three aspects are similar: late start (except in China and Russia), vaccination coverage (complete schedule) under 70% of the population in December 2022 (except for China and Brazil), and vaccine hesitancy. The diverse responses BRICS countries adopted regarding social distancing and vaccination against COVID-19 were related to political, social, and economic factors that conditioned the implementation of such measures.

## Resumen

El objetivo de este estudio fue analizar comparativamente las medidas de distanciamiento social y de vacunación implementadas por los países del BRICS en respuesta a la pandemia de la COVID-19. Se trata de un estudio exploratorio con base en las aportaciones del institucionalismo histórico y del método histórico-comparado en las ciencias sociales. Se dio prioridad a dos ejes: el distanciamiento social y las medidas de vacunación. Parte de los países del BRICS mostraron características similares, tales como la aplicación de medidas de distanciamiento social poco extensas, flexibles y de coordinación descentralizada (con la excepción de China), la debilidad de las medidas de asistencia social (Brasil, India y Sudáfrica) y el uso de recursos biotecnológicos (presentes en China y Rusia). Con respecto a la vacunación, tres aspectos son similares: inicio tardío (excepto en China y Rusia); cobertura de vacunación (calendario completo) de menos del 70% de la población en diciembre de 2022 (excepto en China y Brasil); y vacilación de la vacuna. Las respuestas adoptadas por los países del BRICS en términos de distanciamiento social y de vacunación contra la COVID-19 fueron diversas y relacionadas con factores políticos, sociales y económicos, que condicionaron la implementación de estas medidas.

## Introdução

A pandemia da COVID-19 causou uma crise multidimensional desafiando as capacidades locais, nacionais e globais de preparação e resposta, colocando em evidência, sobretudo, os sistemas de saúde e sua resiliência ^1^. Para Pereira et al. ^2^, a resposta nacional a uma emergência sanitária será condicionada, em grande medida, pelas capacidades internas das nações para desenvolvimento de ações e estratégias em cinco dimensões: governança e coordenação nacional; controle da propagação da epidemia; fortalecimento do sistema de saúde; apoio social e econômico; e comunicação com a sociedade. Neste sentido, desigualdades estruturais e político-insitucionais, iniquidades no financiamento, organização e acesso aos sistemas de saúde, bem como diferenças nas capacidades produtivas e tecnológicas para desenvolvimento de insumos e biofármacos, podem impactar de forma contundente as medidas adotadas frente à COVID-19 e suas consequências sobre a população ^2^ ^,^ ^3^.

Durante a primeira fase da pandemia, as intervenções não farmacêuticas, como o isolamento, a quarentena, o distanciamento social e as medidas de contenção comunitária se configuraram como as principais estratégias de mitigação, visto que não haviam vacinas disponíveis e medicamentos com eficácia cientificamente comprovada contra a COVID-19 ^4^. Alguns dos principais benefícios da adoção destas medidas pelos países foi o ganho de tempo para a organização dos serviços, evitando, em muitos contextos, o colapso dos sistemas de saúde e, por consequência, as mortes decorrentes da falta de assistência aos casos graves ^5^.

Na segunda fase da pandemia, no entanto, com o advento das vacinas contra a COVID-19, os imunizantes se tornaram a ferramenta mais eficaz de combate ao vírus e, após as campanhas vacinais e a ampla imunização da população, muitos estudos evidenciaram o impacto das mesmas na redução do número de internações hospitalares e óbitos ^6^ ^,^ ^7^.

Embora as estratégias de distanciamento social e vacinação tenham se mostrado fundamentais para a contenção da pandemia em diferentes momentos, sendo inclusive listadas como alguns dos objetivos do *COVID-19 Strategy Update* ^8^ - uma recomendação do Comitê de Emergências do Regulamento Sanitário Internacional (RSI) -, a implementação destas estratégias teve inúmeros desafios de ordem social, econômica e política, sobretudo nos países do sul global ^9^, onde se enquadra a maioria dos países do BRICS (Brasil, Rússia, Índia, China e África do Sul), com exceção da Rússia. 

Os países do BRICS assumiram uma grande projeção no cenário mundial de resposta à crise sanitária em relação às medidas de distanciamento social e vacinação. A Índia foi crucial para o fornecimento dos imunizantes através do Serum Institute; China e Rússia despontaram no uso da biotecnologia, liderando o desenvolvimento de vacinas contra a COVID-19, além de utilizarem destes recursos para promover estratégias de distanciamento social; o Brasil assumiu um protagonismo na produção de insumos farmacêuticos ativos e vacinas contra a COVID-19 por meio de Bio-Manguinhos/FIOCRUZ e do Instituto Butantan, bem como pela capacidade de distribuição nacional através do Sistema Único de Saúde (SUS); e a África do Sul também se destacou na instauração de medidas de distanciamento social de acordo com a incidência da doença no país ^10^ ^,^ ^11^ ^,^ ^12^. 

Apesar desses esforços, os países do BRICS foram fortemente afetados pela COVID-19, estando, em diferentes momentos, entre aqueles com maior número de casos ou óbitos ^13^. Diante disso, este estudo objetivou analisar, de forma comparada, as medidas de distanciamento social e vacinação implementadas por estes países em resposta à pandemia da COVID-19.

## Metodologia

Trata-se de um estudo exploratório ancorado nas contribuições do institucionalismo histórico ^14^ e do método histórico-comparado em ciências sociais ^15^. Baseando-se em modelos de análise da resposta dos sistemas de saúde à COVID-19 desenvolvidos previamente ^2^ ^,^ ^3^, dois eixos foram priorizados: as medidas de distanciamento social e de vacinação, dada sua importância reconhecida para enfrentamento da COVID-19 e proteção da população mais vulnerabilizada ^8^.

Os países do BRICS foram selecionados como casos de análise por serem considerados potências emergentes, que tiveram relevância estratégica durante a pandemia por sua capacidade tecnológica e de fornecimento de imunizantes; com número de casos e óbitos por COVID-19 expressivos; com sistemas de saúde baseados em diferentes modelos; e com capacidades de resposta variáveis à crise sanitária quanto ao distanciamento social e à vacinação. Por se tratar de um estudo que teve como recorte temporal o período em que a COVID-19 foi reconhecida como pandemia pela Organização Mundial da Saúde (OMS), a coleta de dados foi delimitada entre março de 2020 e maio de 2023. 

O Quadro 1 descreve as etapas, objetivos e componentes vinculados a cada um dos eixos de análise, bem como as estratégias e fontes de pesquisa utilizadas. Na primeira etapa, cada caso foi estudado em profundidade ^15^, sendo identificadas as principais características das medidas de distanciamento social e vacinação em cada país, a partir de análise documental, bibliográfica e de dados secundários. Os dados relacionados à mobilidade populacional e cobertura vacinal foram tratados em Excel (https://products.office.com/) por meio de estatística descritiva. A segunda etapa consistiu da análise comparada entre os achados, destacando semelhanças, diferenças e fatores que possam colaborar para compreensão das mesmas.

**Quadro 1:** Matriz de análise das medidas de distanciamento social e vacinação.

ETAPAS E OBJETIVOS ESPECÍFICOS	COMPONENTES DE ANÁLISE	VARIÁVEIS DE ANÁLISE	ESTRATÉGIAS DE PESQUISA E FONTES CONSULTADAS
1ª Etapa: Analisar as medidas de distanciamento social e vacinação implementadas pelos países do Brics entre março de 2020 e maio de 2023	Distanciamento social	- Início da implementação das medidas; - Mobilidade populacional (adesão); - Características gerais das medidas	(1) Análise de informações e documentos disponíveis nos sítios oficiais do Ministério da Saúde ou órgãos vinculados, dos países do Brics: Ministério da Saúde do Brasil (https://www.gov.br/saude/pt-br) Ministério da Saúde da Federação Russa (Rozminzdrav): (https://edu.rosminzdrav.ru/covid-19/) Ministry of Health and Family Welfare (https://mohfw.gov.in/) National Health Commission of the People’s Republic of China (China NHC) (http://en.nhc.gov.cn/publications.html) National Department of Health od South Africa (https://www.health.gov.za/) (2) Análise de dados secundários disponíveis em base de comparação internacional (https://ourworldindata.org/coronavirus) (3) Análise de artigos científicos disponíveis em bases indexadas nacionais e internacionais (BVS, Scielo, PubMed e Web of Science). Busca não-sistemática dirigida pelos objetivos específicos a fim de orientar a discussão dos resultados e análise comparada. Levantamento realizado entre julho 2023 e abril 2024
	Vacinação contra a COVID-19	- Início da campanha vacinal; - Cobertura vacinal; - Adesão populacional e hesitação vacinal; - Características gerais das medidas.	
2ª Etapa: Discutir os fatores que condicionaram a adoção de medidas de distanciamento social e vacinação nos países do BRICS	Condicionantes das medidas de distanciamento social e vacinação frente à COVID-19 nos países do BRICS	- Semelhanças e diferenças entre as medidas adotadas pelos países do BRICS; - Fatores que condicionaram as medidas em cada caso.	

Fonte: elaborado pelos autores.

Como o estudo faz uso exclusivamente de dados secundários e de acesso livre, não foi necessária sua submissão ao Comitê de Ética em Pesquisa (CEP).

## Resultados

As medidas de distanciamento social e de vacinação adotadas por cada um dos países analisados têm relação com o quadro epidemiológico vivenciado por eles nos diferentes momentos da pandemia de COVID-19. Os registros de primeiro caso e primeiro óbito por COVID-19 foram na China. Índia e Rússia foram o segundo e terceiro país dos BRICS, respectivamente, a identificar casos de COVID-19. Brasil e África do Sul foram os últimos a registrarem casos. Quanto aos óbitos, Índia e Brasil foram os segundos, seguidos por Rússia e África do Sul. Entre janeiro de 2020 e maio de 2023, o Brasil foi o país com maior número de casos acumulados por milhão (178.254.060), seguido pela Rússia (156.966.800), África do Sul (64.993.727), China (69.638.510), e Índia (31.531.260). No que se refere ao número de óbitos acumulados por milhão, neste mesmo período, o Brasil também ocupou o primeiro lugar (3.337.193), seguido pela Rússia (2.727.500), África do Sul (1.644.719), Índia (372.895) e China (84.912) ^13^. A Tabela 1 apresenta uma síntese dessa situação.

**Tabela 1:** Datas do primeiro caso e primeiro óbito, número de casos e número de óbitos por COVID-19 nos países dos BRICS, janeiro de 2020 a maio de 2023.

Indicadores	China	Índia	Rússia	Brasil	África do Sul
Data do primeiro caso	17/Nov/2019	30/Jan/2020	31/Jan/2020	26/Fev/2020	01/Mar/2020
Data do primeiro óbito	02/Jan/2020	12/Mar/2020	19/Mar/2020	12/Mar/2020	27/Mar/2020
Número de casos acumulados (por milhão de pessoas) por fase pandêmica	1ª Fase: 126,78	1ª Fase: 7.694,85	1ª Fase: 6.843,36	1ª Fase: 25.621,51	1ª Fase: 11.535,33
	2ª Fase: 6.611,19	2ª Fase: 16.818,93	2ª Fase: 29.149,78	2ª Fase: 76.624,22	2ª Fase: 14.368,83
	3ª Fase: 62.861,07	3ª Fase: 5.936,31	3ª Fase: 38.661,6	3ª Fase: 40.832,25	3ª Fase: 24.527,85
	-	4ª Fase: 28.961,43	4ª Fase: 52.923,1	4ª Fase: 19.638,15	4ª Fase: 12.018,41
	-	-	5ª Fase: 22.818,08	5ª Fase: 9.795,76	5ª Fase: 4.470,67
	-	-	6ª Fase: 7.510,83	-	6ª Fase: 792,94
Total de casos acumulados (por milhão de pessoas)	69.638.510	31.531.260	156.966. 800	178.254.060	64.993.727
Número de óbitos acumulados (por milhão de pessoas) por fase pandêmica	1ª Fase: 4,05	1ª Fase: 109,83	1ª Fase: 118,12	1ª Fase: 752,46	1ª Fase: 335,94
	2ª Fase: 16,88	2ª Fase: 227,05	2ª Fase: 713,90	2ª Fase: 2.105,7	2ª Fase: 548,19
	3ª Fase: 63,86	3ª Fase: 33,16	3ª Fase: 1326,48	3ª Fase: 236,49	3ª Fase: 617,94
		4ª Fase: 4,73	4ª Fase: 414,22	4ª Fase: 107,39	4ª Fase: 172,85
		-	5ª Fase: 81,1	5ª Fase: 52,08	5ª Fase: 31,49
		-	6ª Fase: 35,06	-	6ª Fase: 10,24
Total de óbitos acumulados (por milhão de pessoas)	84.912	372.895	2.737.500	3.337.193	1.644.719

Fonte: Mathieu et al. ^13^.

Observa-se que, temporalmente, as fases da pandemia foram bastante distintas entre os BRICS, de modo que os picos de casos e óbitos ocorreram em momentos diferentes, guardando relação com as ações de distanciamento físico e vacinação implementadas nacionalmente e a capacidade de cada país em sustentá-las.

### Medidas de distanciamento social

A China se destaca, entre os BRICS, como o país que implementou medidas mais rigorosas e mais duradouras de distanciamento físico. As primeiras iniciativas de *lockdown* no país foram implementadas em 23 de janeiro de 2020 na cidade de Wuhan, e no dia seguinte estendidas para outras 15 cidades da província de Hubei. Além disso, o feriado nacional de celebração do Ano Novo Lunar, que ocorreu entre os dias 25 a 31 de janeiro, foi estendido até 10 de fevereiro ^16^. As restrições implementadas foram rigorosas e incluíam sistemas de monitoramento como drones; aplicativos para entrar em lugares públicos; e reconhecimento facial nas ruas ^12^ ^,^ ^17^. Em agosto de 2021, a China implementou a política de distanciamento social “*Dynamic zero-COVID*”, caracterizada por política de medidas rígidas de *lockdown*, que permitiu ao país um maior controle da pandemia em relação a outros países ^18^.

O Brasil foi o segundo país dos BRICS a implementar medidas de distanciamento social. Tiveram início em 11 de março de 2020 por meio de iniciativas autônomas das Unidades Federadas, com níveis de rigor variados, visto que não houve uma coordenação federativa voltada a todo território nacional. Os estados brasileiros apresentaram níveis altos e sustentados de distanciamento social até o final do mês de março de 2020, a despeito das pressões exercidas por associações de classe de empresas, e até mesmo discursos de autoridades do governo que defendiam menor rigor destas normas ^19^ ^,^ ^20^. O Brasil enfrentou dificuldades de ordem econômica e social na implementação das medidas de distanciamento social, visto que uma parcela considerável da população exposta a vínculos de trabalho frágeis não teve a opção de adotar o trabalho remoto, além do auxílio insuficiente ofertado pelo governo brasileiro ^19^.

O terceiro país a implementar medidas de distanciamento social foi a África do Sul, em 23 de março. Foi adotado um sistema de níveis de restrição baseado em cinco estágios, que contemplava desde um *lockdown* (nível 5) até a retomada da maioria das atividades, com precauções e orientações de saúde seguidas em todos os momentos (nível 1) ^21^. No entanto, o governo sul-africano foi promovendo paulatinamente medidas de flexibilização devido às pressões relacionadas à pobreza e desemprego no país ^22^ ^,^ ^23^. 

Na Índia, o governo decretou um *lockdown* em 24 de março e este perdurou até 31 de maio, sendo o quarto país dos BRICS a instituir tais medidas. Durante esse período, o governo indiano dividiu os distritos em três zonas com base na propagação do vírus - zona verde, sem casos; zona laranja, com registro de poucos casos; e zonas vermelhas, consideradas focos de disseminação. Esta classificação passou a ser utilizada como critério para a implementação das medidas de distanciamento social em cada região, descentralizando a implementação das mesmas. Contudo, a eficácia das medidas introduzidas foi limitada por questões sociais e econômicas ^24^ ^,^ ^25^ ^,^ ^26^. Soma-se ao contexto socioeconômico, as posturas controversas do governo indiano, que desencorajou o distanciamento social em prol de questões políticas e religiosas, como as eleições estaduais e o Kumbh Mela, que atraiu cerca de 3,5 milhões de devotos indianos ^27^.

O último dentre os BRICS a implantar medidas de distanciamento social foi a Rússia. Tais medidas foram instauradas pelo Governo Federal em 25 de março de 2020, baseando-se em um recesso de 30 de março a 3 de abril. Na mesma data, foi implantado o *lockdown* em Moscou e em outras regiões do país ^28^. A Rússia lançou mão de recursos tecnológicos para promover o distanciamento social, como: passes digitais para verificação do movimento de cidadãos via aplicativo; o programa *Moscow Safe City* [Moscou Cidade Segura], de câmeras de reconhecimento facial em espaços públicos; e o uso de drones pela Guarda Nacional Russa para controle da circulação de pessoas ^11^. A partir do dia 9 de junho de 2020, o governo de Moscou anunciou o início das medidas de flexibilização mediante a queda no número de novos casos diários no país ^29^. Ressalta-se ainda que, as medidas de distanciamento social foram transferidas aos governos regionais, provocando uma resposta subnacional variável e descoordenada à crise sanitária ^28^.

Considerando o período de março de 2020 a julho de 2022, Brasil, Índia e África do Sul apresentaram uma queda acentuada nos níveis de mobilidade nas estações de transporte público e locais de trabalho. Nota-se que, nos três casos, no início da pandemia (março a julho de 2020), a queda da mobilidade foi mais acentuada, refletindo um maior rigor das medidas de distanciamento social naquele primeiro momento. Ao longo do tempo, no entanto, estes níveis apresentaram tendência de crescimento (Quadro 2). Não havia dados disponíveis para China e Rússia.

**Quadro 2:** Síntese das características e condicionantes das medidas de distanciamento social durante a pandemia da COVID-19 nos países do BRICS.

DISTANCIAMENTO SOCIAL					
	CHINA	BRASIL	ÁFRICA DO SUL	ÍNDIA	RÚSSIA
Início da implementação	23/Jan/2020	11/Mar/2020	23/Mar/2020	24/Mar/2020	25/Mar/2020
Mobilidade (%) medida nas estações de transporte público *	n/d	Mar/2020: -60,71 Mar/2021: -34,43 Mar/2022: 2	Mar/2020: -63,14 Mar/2021: -23,43 Mar/2022: 17	Mar/2020: -2,86 Mar/2021: -0,29 Mar/2022: 23,29	n/d
Mobilidade (%) medida nos locais de trabalho *	n/d	Jul/2020: -45,57 Jul/2021: -15,43 Jul/2022: 30,71	Mar/2020: -40,14 Mar/2021: -11,57 Mar/2022: 10,14	Mar/2020: -65,86 Mar/2021: -19,43 Mar/2022: 25,43	n/d
Características das medidas de distanciamento social	Medidas de distanciamento social coordenadas nacionalmente, duradouras e acompanhadas de auxílio governamental ^17^ Adoção de medidas rígidas de *lockdown*, denominada “*Dynamic zero-Covid*” ^19^ Extenso uso de tecnologias digitais, incluindo sistemas de monitoramento de sintomas por aplicativos e testes; e controle da circulação das pessoas por drones e reconhecimento facial ^17^	Medidas de distanciamento social sob coordenação descentralizada, de extensão variável entre estados e municípios, e acompanhadas de auxílio governamental insuficiente ^20^ ^,^ ^21^ Adoção de medidas flexíveis e descoordenadas no contexto federativo, mesmo em momentos críticos (de incidência e mortalidade crescentes) ^20^ Uso de medidas tradicionais de distanciamento social (controle de fronteiras e restrições no uso do espaço público) ^20^ ^,^ ^21^	Medidas de distanciamento social coordenadas nacionalmente, pouco extensas e acompanhadas de auxílio governamental insuficiente ^22^ ^,^ ^23^ ^,^ ^24^ Adoção de medidas flexíveis, que variaram do *lockdown* (nível 5) à retomada da maioria das atividades (nível 1) ^22^ Uso de medidas tradicionais de distanciamento social (controle de fronteiras e restrições no uso do espaço público) ^22^	Medidas de distanciamento social sob coordenação descentralizada, pouco extensas e acompanhadas de auxílio governamental insuficiente ^25^ ^,^ ^26^ ^,^ ^27^ Adoção de medidas flexíveis, com base na divisão dos distritos em três zonas (conforme nível de disseminação da COVID-19) ^25^ ^,^ ^26^ ^,^ ^27^ Uso de medidas tradicionais de distanciamento social (controle de fronteiras e restrições no uso do espaço público) ^25^ ^,^ ^26^ ^,^ ^27^	Medidas de distanciamento social pouco extensas e sob coordenação descentralizada ^29^ Adoção de medidas rigorosas na primeira fase da pandemia, flexibilizadas nas demais ^29^ Extenso uso de tecnologias digitais, como passes digitais para liberação da circulação de pessoas; e implementação do programa Moscow Safe City, com base em câmeras de reconhecimento facial em espaços públicos e uso de drones ^11^.
Condicionantes das medidas de distanciamento social	Presença de liderança nacional, com alto investimento do governo central em políticas sanitárias e sociais para resposta à COVID-19 ^17^ Capacidade industrial nacional alta nas áreas biotecnológica e de tecnologia da informação, coordenada pelo governo central (por meio de parcerias e governança multinível) para apoiar as medidas de distanciamento social ^17^	Ausência de liderança nacional e investimentos insuficientes em medidas sociais de resposta à COVID-19 ^20^ ^,^ ^21^ Presença de um sistema universal de saúde com um arcabouço de gestão federativa subaproveitado em função da postura negacionista do Governo Federal ^20^ ^,^ ^21^ Dificuldades de ordem econômica e social (pobreza, informalidade no trabalho e populações vulnerabilizadas) ^21^	Presença de liderança nacional, mas investimentos insuficientes em medidas sociais de resposta à COVID-19 ^23^ ^,^ ^24^ Ausência de um sistema de saúde universal e de capacidade nacional na produção de testes e biofármacos ^23^ ^,^ ^24^ Dificuldades de ordem econômica e social (aumento da pobreza e desemprego no país) ^23^ ^,^ ^24^	Ausência de liderança nacional e investimentos insuficientes em medidas sociais de resposta à COVID-19 ^25^ ^,^ ^26^ ^,^ ^27^ Gestão fragmentada, com posturas controversas do governo indiano, que desencorajou o distanciamento social e não atuou de modo a direcionar a capacidade nacional de produção de testes para atender a demanda nacional prioritariamente ^28^ Dificuldades de ordem social e econômica ^25^ ^,^ ^26^ ^,^ ^27^	Ausência de liderança nacional e investimentos insuficientes em medidas sociais de resposta à COVID-19 ^29^ Gestão fragmentada, com subaproveitamento da estrutura federativa do país e do sistema nacional de saúde para gestão da pandemia ^29^ Capacidade nacional alta no uso de tecnologias digitais, aplicadas pelo Governo Federal para implementação de medidas de distanciamento social nacionalmente ^11^

n/d: dados não disponíveis.

Fonte: Mathieu et al. ^13^. Dados referentes a março/2020 a julho/2022.

* Mobilidade (%).

### Vacinação

A Rússia se destacou por ser o primeiro país do mundo a anunciar um imunizante contra a COVID-19 em 11 de agosto de 2020, a vacina Sputnik V, desenvolvida pelo Gamaleya Research Institute em Moscou. Desde o anúncio, a vacina russa levantou uma série de questionamentos relativos à sua segurança e eficácia, uma vez que a mesma foi anunciada antes do início do ensaio da fase III ^30^. A este respeito, destaca-se que foram identificadas falhas no desenvolvimento do produto nas etapas dos estudos clínicos (fases 1, 2 e 3), além da ausência ou insuficiência de dados de controle de qualidade, segurança e eficácia ^31^. Foi o primeiro país dos BRICS a dar início a campanha de vacinação contra a COVID-19, em 5 de dezembro de 2020, e enfrentou baixa adesão, de modo que o governo de Moscou implementou medidas como a concessão de incentivos financeiros à população ^32^ ^,^ ^33^. A baixa adesão à campanha de vacinação na Rússia se justificou sobretudo pelo ceticismo da população em relação à segurança e eficácia das vacinas desenvolvidas no país; pela crença nos efeitos colaterais das mesmas; e pela desaprovação ao Governo Federal ^34^.

A China foi o segundo país dos BRICS a iniciar a vacinação contra COVID-19, em 15 de dezembro de 2020. O país concedeu aprovação de uso emergencial para sete vacinas da COVID-19 ^35^. A campanha vacinal contou com divulgação oficial e liderança do governo central, gerando alta cobertura populacional. Um dos principais desafios enfrentados pela China foi a adesão à vacinação pela população idosa. A este respeito, Yuan ^36^ afirmou que, entre os cidadãos maiores de 80 anos, pouco mais da metade havia recebido duas doses e menos de 20% receberam um reforço. Um fator preponderante para este cenário foi a hesitação vacinal, fomentada pelo senso coletivo de falta de urgência em ser vacinado, sobretudo pelo rigor das medidas de distanciamento social. 

Na Índia, a campanha de vacinação contra a COVID-19 foi iniciada em 16 de janeiro de 2021. A despeito de sediar um dos maiores produtores mundiais de vacinas, o Instituto Serum, além de outros grandes centros biotecnológicos de produção de vacinas e medicamentos como a Bharat Biotech e a Indian Immunologicals, o país enfrentou dificuldades de aquisição de imunizantes para atender sua demandas internas, além de dificuldades logísticas, tais como distribuição inadequada de doses de imunizantes; um planejamento ineficiente na aquisição prévia dos mesmos, levando-se em consideração as expectativas de abastecimento do mercado interno e externo; além da cobrança pela administração das vacinas no setor público e privado ^37^ ^,^ ^38^ ^,^ ^39^. Destaca-se ainda que a Índia enfrentou um forte movimento de hesitação vacinal associada à desinformação, sobretudo na zona rural, com crenças de que a vacina poderia alterar o ciclo menstrual e reduzir a fertilidade ^38^. 

O Brasil iniciou sua campanha de vacinação contra a COVID-19 em 18 de janeiro de 2021 ^40^, quase um mês depois de seus vizinhos na América Latina, e este atraso foi justificado por uma estratégia vacinal marcada por conflitos políticos. Desde o início da pandemia, o Governo Federal criou crises diplomáticas com a China e a Índia, afetando a capacidade de produção de vacinas pelo Instituto Butantan e a Fiocruz ^41^. Ressalta-se ainda a letargia do governo mediante as negociações de compra dos imunizantes, como ocorreu em 2020, via Aliança Mundial de Vacinas (*COVAX Facility*), com aquisição de doses insuficientes até mesmo para os grupos prioritários, além da inação diante das negociações de compra dos insumos, como ocorreu com a Pfizer ^42^. O Brasil também vivenciou uma hesitação vacinal, impulsionada, em parte, pelas declarações controversas e negacionistas do presidente da república em relação às vacinas ^43^. Apesar disso, o Brasil atingiu uma alta cobertura vacinal. Cabe ressaltar o papel do SUS, a capilaridade da atenção primária (presente em todos os 5.570 municípios) e a organização do Programa Nacional de Imunizações (que compreende um dos maiores e mais completos programas de vacinação do mundo), que atuaram como fatores decisivos para viabilizar a distribuição das doses da vacina para todo território nacional ^44^.

Último dentre os BRICS, a África do Sul iniciou seu programa nacional de vacinação contra a COVID-19 em 17 de fevereiro de 2021, com importantes acordos e negociações para aquisição de imunizantes com a Johnson & Johnson, Pfizer, Sinovac, Sinopharm e Sputnik V, além de um acordo com a *COVAX Facility* e União Africana. No entanto, embora o país tenha apresentado um desempenho melhor em sua campanha de vacinação em relação aos outros países da África subsaariana, o país não atingiu sua meta de imunizar 67% da sua população até o final do ano de 2021. Esse resultado pode ser relacionado a diversos fatores: dificuldade de aquisição de imunizantes; falta de solidariedade dos países do norte global no compartilhamento de vacinas da COVID-19 e de tecnologias para a produção independente das mesmas pelos países do sul ^45^; e o forte movimento de hesitação vacinal, por descontentamento com o governo e por questionamentos quanto à segurança das vacinas ^13^ ^,^ ^21^ ^,^ ^46^. 

Em dezembro de 2022, o país do BRICS com melhor desempenho em termos de cobertura vacinal foi a China (89,35% da população com esquema vacinal completo). O segundo melhor foi o Brasil (81,22% da população com esquema vacinal completo); em seguida, a Índia (67,12% da população com esquema vacinal completo); posteriormente, a Rússia (54,54% da população com esquema vacinal completo); e, por fim, a África do Sul (35,13% da população com esquema vacinal completo) ^13^ (Quadro 3).

**Quadro 3:** Síntese das características e condicionantes das medidas de vacinação durante a pandemia da COVID-19 nos países do BRICS.

VACINAÇÃO					
	CHINA	BRASIL	ÁFRICA DO SUL	ÍNDIA	RÚSSIA
Início da campanha vacinal	15/Dez/2020	18/Jan/2021	17/Fev/2021	16/Jan/2021	5/Dez/2020
COBERTURA VACINAL (%) *					
Esquema completo	89,35	81,22	35,13	67,12	54,54
Parcialmente vacinados	91,68	87,57	40,02	72,48	60,58
Doses de reforço	57,21	56,95	6,47	15,71	13,49
Características das medidas de vacinação	Rápido início da campanha vacinal ^36^ Adesão populacional alta (89% da população com esquema completo em dezembro de 2022) ^13^ Hesitação vacinal, sobretudo entre os maiores de 80 anos ^37^	Início tardio da campanha vacinal ^41^ Adesão populacional alta (81% da população com esquema completo em dezembro de 2022) ^13^ Hesitação vacinal, em um contexto marcado por conflitos políticos ^44^	Início tardio da campanha vacinal ^46^ Adesão populacional baixa (35% da população com esquema completo em dezembro 2022) ^13^ Hesitação vacinal, em um contexto de dificuldade de aquisição de imunizantes ^13^ ^,^ ^22^ ^,^ ^47^	Início tardio da campanha vacinal ^38^ ^,^ ^39^ ^,^ ^40^ Adesão populacional baixa (67% da população com esquema completo em dezembro 2022) ^13^ Hesitação vacinal, em um contexto de dificuldade de aquisição de imunizantes para atender as demandas internas do país; e de dificuldades logísticas para realizar a vacinação ^39^	Rápido início da campanha vacinal ^31^ Adesão populacional baixa (54,5% da população com esquema completo em dezembro 2022) ^13^ Hesitação vacinal, mesmo diante da implementação de incentivos financeiros pelo governo federal para ampliar a vacinação ^35^
Condicionantes das medidas de vacinação	Capacidade de produção e compra de vacinas alta, favorecida por uma postura pró-vacinação do governo central ^17^ Divulgação nacional da campanha de vacinação por meio de canais oficiais do governo, contribuindo para a confiança e adesão populacional ^17^ Hesitação vacinal relacionada a um senso coletivo de falta de urgência em ser vacinado, sobretudo pelo rigor das medidas de distanciamento social ^37^	Letargia do governo federal nas negociações relacionadas a insumos e vacinas, afetando a capacidade de produção nacional ^43^ Postura negacionista do governo federal frente à COVID-19 e à vacinação ^44^ Positivamente, destaca-se o SUS e a capilaridade da APS em todos os municípios do país, fator que favoreceu a vacinação ^45^ Hesitação vacinal relacionada a *fake news* favorecidas por declarações controversas do presidente da República ^44^	Capacidade de produção e compra de vacinas baixa, em um contexto de falta de solidariedade no compartilhamento de vacinas e de tecnologias para a produção independente das mesmas pelos países do sul global ^46^ Sistema de saúde segmentado e fragmentado que não favoreceu a distribuição das vacinas ^46^ Hesitação vacinal relacionada ao descontentamento com o governo e pela disseminação de notícias falsas referentes à segurança das vacinas ^13^ ^,^ ^22^ ^,^ ^47^	Capacidade de produção de vacinas alta, contudo, ausência de uma política do governo para garantia da cobertura da população nacional ^29^ ^,^ ^38^ ^,^ ^40^ Planejamento ineficiente na aquisição prévia de vacinas pelo governo ^38^ ^,^ ^39^ ^,^ ^40^ Sistema de saúde segmentado e fragmentado que não favoreceu a distribuição das vacinas; e cobrança pela administração das vacinas no setor público e privado ^38^ ^,^ ^39^ ^,^ ^40^ Hesitação vacinal relacionada à desinformação da população ^39^	Capacidade de produção e compra de vacinas alta, contudo, campanha vacinal desfavorecida pela desaprovação ao Governo Federal ^33^ ^,^ ^34^ Hesitação vacinal relacionada ao ceticismo da população frente às vacinas desenvolvidas no país em função da crença nos efeitos colaterais das mesmas e da baixa confiança no Governo Federal ^35^

APS: atenção primária à saúde; SUS: Sistema Único de Saúde.

Fonte: cobertura vacinal: Mathieu et al. ^13^. Dados referentes a dezembro de 2022.

Embora a campanha de vacinação contra a COVID-19 na China tenha sido bem sucedida em termos de cobertura vacinal, ao final do ano de 2022, o país passou a registrar um aumento no número de casos e óbitos pela doença. Este cenário epidemiológico foi associado a três fatores: o fim da política “*Dynamic zero-COVID*” de *lockdown* rigoroso ^47^; a baixa adesão à vacinação pela população idosa ^35^; e a baixa cobertura de doses de reforço ^47^. No Brasil e na Índia, o avanço da cobertura vacinal refletiu a redução drástica do número de casos e óbitos por COVID-19 entre as segunda e terceira fases pandêmicas. Na Rússia e África do Sul, a lentidão na cobertura vacinal acarretou em uma redução mais lenta no número de casos e óbitos pela COVID-19, que passou a ser mais expressiva a partir da quarta fase pandêmica nesses países (Tabela 1).

Os Quadros 2 e 3 sintetizam as principais características das medidas de distanciamento social e vacinação nos países do BRICS. Além disso, sumarizam alguns fatores que condicionaram a implementação destas medidas em cada país, afetando sua capacidade de resposta à COVID-19.

## Discussão

A análise das medidas de distanciamento social e vacinação revelaram mais diferenças do que semelhanças nas respostas à pandemia entre os países do BRICS, se assemelhando a outros resultados evidenciados pela literatura ^48^ ^,^ ^49^. Algumas das características semelhantes identificadas em parte dos países do bloco foram: a adoção de medidas de distanciamento social pouco extensas, flexíveis e sob coordenação descentralizada (com exceção da China); a fragilidade das medidas de auxílio social (comum nos casos do Brasil, Índia e África do Sul); e o uso de recursos biotecnológicos e de tecnologias digitais (presentes na China e Rússia). No que tange à vacinação, três aspectos se assemelham: início tardio (exceto na China e Rússia); cobertura vacinal (esquema completo) inferior a 70% da população em dezembro de 2022 (exceção para China e Brasil); e hesitação vacinal, vivenciada nos cinco países, ainda que em diferentes proporções. 

Destacando medidas exitosas, o uso intensivo de recursos biotecnológicos e tecnologias digitais na China e Rússia aparece como um fator importante, que condicionou favoravelmente a resposta desses países. Tais estratégias também foram usadas por outras nações, como o Irã, que utilizou o aplicativo Mask, e a Alemanha, com o aplicativo Corona-Warn ^50^. 

A sustentabilidade e a eficácia das medidas de distanciamento social dependem do estabelecimento de políticas de proteção social e de apoio às populações vulnerabilizadas ^51^. Sustentando esta premissa, um estudo realizado no Brasil identificou que quanto mais a população teve oportunidade de incorporar-se ao trabalho remoto, maior foi o nível de distanciamento físico. Por outro lado, quanto maior a proporção de trabalho informal, menor foi a magnitude do distanciamento social ^52^. África do Sul, Índia e Brasil têm em comum a implementação de auxílios sociais insuficientes frente à COVID-19, em um contexto de grande parcela da população com vínculo de trabalho informal (em 2022, esta parcela na África do Sul e no Brasil era 45% e 40%, respectivamente, e 70% na Índia) ^53^ ^,^ ^54^ ^,^ ^55^. 

A hesitação vacinal apresentou diferentes condicionantes em cada país. No Brasil, foi associada à postura negacionista do chefe do executivo ^43^; na Rússia, à desconfiança com a segurança das vacinas e à desaprovação do governo pela população ^34^; na Índia, à falta de conhecimento da população ^38^; na China, à falsa noção de segurança associada ao rigoroso distanciamento social, sobretudo entre os idosos ^36^; e na África do Sul, à falta de conhecimento da população, desaprovação do governo e descrença no acesso equitativo das mesmas ^46^. 

Estes fatores corroboram os aspectos indicados pela *Sage Working Group on Vaccine Hesitancy* ^56^ como condicionantes do aceite de uma vacina pela população: complacência, confiança e conveniência. A complacência se refere à suposição de que o risco de contrair uma doença específica é baixo e, portanto, que a vacinação não é essencial e pode ser evitada, como foi observado no caso da China. A confiança se refere à convicção da eficácia e segurança das vacinas, no sistema que as administra e nos decisores políticos, o que foi seriamente afetado em países como o Brasil, Rússia, Índia e África do Sul; e a conveniência se relaciona à acessibilidade às vacinas, o que não se concretizou, sobretudo na África do Sul ^56^.

As diferenças nas medidas de distanciamento social e vacinação estão relacionadas a dois condicionantes principais: a liderança nacional da gestão da pandemia, fragmentada no Brasil, Rússia e Índia, em contraponto à resposta coordenada da China; e a capacidade de produção de vacinas contra a COVID-19, destacada na China, Rússia e Índia, em contraste à falta de infraestrutura para a produção e barreiras de acesso aos imunizantes na África do Sul.

A gestão fragmentada das medidas de distanciamento social e vacinação, vivenciada pelo Brasil, Rússia e Índia, também foi documentada em países como Itália e Estados Unidos ^57^ ^,^ ^58^. Como consequência, estes países enfrentaram crises políticas na gestão da pandemia, com ações desconexas e comunicação falha intra-governo e com a sociedade. A este respeito, Bargain & Aminjonov ^59^ argumentam que a confiança do público na capacidade do governo é crucial, uma vez que esta confiança apoia as atitudes e o comportamento público em momentos críticos. 

Neste sentido, a China demonstrou uma resposta mais exitosa em relação aos outros países do Brics, com uma gestão coordenada nacionalmente e com integração de políticas sociais e de saúde ^44^. A este respeito, Baker et al. ^60^ relataram que os países mais bem sucedidos na condução da crise sanitária foram aqueles que adotaram medidas assertivas e coordenadas de *lockdown* associadas à forte vigilância epidemiológica, com busca ativa de casos, controle de contatos, isolamento e testagem massiva, como foi o caso da China. 

Outra diferença também se deu a partir do maior destaque alcançado pela China, Índia e Rússia na autossuficiência na produção dos imunizantes, condicionada pelos recursos biotecnológicos destes países. Além disso, China e Índia foram responsáveis pelo fornecimento de cerca de 14% do total de doses concedidas à *COVAX* até o início de 2022 ^61^. Esta realidade foi altamente contrastada pelo cenário vivenciado pela África do Sul, que enfrentou dificuldades de aquisição e produção de vacinas. 

Alguns fatores podem ser associados a essas semelhanças e diferenças. Como identificado por Pereira et al. ^2^, condicionantes estruturais atuam decisivamente na capacidade de implementação do distanciamento social e da vacinação. Os autores ressaltam o peso de fatores como a capacidade fiscal e financeira dos Estados; cobertura e escopo das políticas de proteção social; e capacidade produtiva nacional-biotecnológica, de equipamentos e tecnologias digitais. Nesse sentido, China e África do Sul localizam-se em extremos opostos.

Da mesma forma, condicionantes políticos desfavoráveis podem fazer com que fatores-chave sejam subaproveitados, não atuando como poderiam para favorecer o êxito das medidas analisadas ^2^ ^,^ ^3^. Esse aspecto é convergente nos casos da Rússia, Índia e Brasil. Na Rússia e Índia, a coordenação fragmentada das medidas de distanciamento social e de vacinação reduziu o potencial de impacto da capacidade de produção local de vacinas; e no Brasil, a descoordenação nacional prejudicou a gestão federativa da pandemia por meio das estruturas do SUS e atrasou a compra e produção de insumos e vacinas. 

Em suma, a análise considera o fato de que estes países do Brics não partem de um mesmo patamar, apresentando diferenças marcantes em seus indicadores socioeconômicos, estrutura de gastos públicos e sistemas políticos. A capacidade de produção e obtenção das vacinas, que se tornou um símbolo geopolítico de prestígio, status e liderança, expôs o poderio tecnológico de países como China e Rússia e, em contraponto, a falta de infraestrutura de países como a África do Sul ^62^. A coordenação nacional e a integração entre políticas sociais e sanitárias favoreceram medidas de distanciamento social sustentadas por um longo tempo, como na China ^63^. Ao passo que a insuficiência de políticas de auxílio social no contexto da pandemia acirrou a crise e as desigualdades em países como África do Sul, Índia e Brasil, situação agravada pela ausência de liderança nacional da resposta à COVID-19 e coordenação fragmentada da mesma. 

Por fim, destaca-se que a análise do desempenho dos países do BRICS, com relação à adoção de medidas de distanciamento social e vacinação, desvelou importantes implicações para a efetividade das políticas públicas de saúde, uma vez que evidenciou a relevância dos condicionantes políticos, sociais e econômicos para que as mesmas sejam bem sucedidas. Dessa forma, ficou explícita a necessidade de que os governos nacionais adotem sistemas de governança eficientes e superem as restrições fiscais-financeiras, a pobreza e alta informalidade no trabalho para que haja credibilidade das populações e viabilidade quanto à execução destas políticas. Ademais, ressalta-se que este estudo apresentou como ponto forte o ineditismo ao focar na análise das medidas de distanciamento social e vacinação para analisar e comparar o desempenho dos países do BRICS durante a pandemia. Uma das limitações do estudo foi o acesso desproporcional a documentos oficiais dos governos, de acordo com a disponibilidade de cada país.

## Conclusões

As respostas adotadas pelos países do BRICS em termos de distanciamento social e vacinação contra a COVID-19 foram diversas. Elas guardam relação com fatores políticos, sociais e econômicos destes países, que condicionaram a implementação de tais medidas, favorecendo-as ou não, a depender do contexto. A influência desses condicionantes evidencia a complexidade da preparação e resposta a emergências sanitárias; aponta para a relevância do papel dos Estados nacionais na construção de estratégias de soberania sanitária; e chama atenção para a necessidade de fortalecer a cooperação entre os países do BRICS na perspectiva da equidade. Destaca-se a importância de se realizar estudos futuros que corroborem a associação entre melhores indicadores socioeconômicos e políticos e resultados mais satisfatórios em termos de políticas públicas entre os países do BRICS.
